# Modelling the monstrosities: experimental and computational systems for studying polyploid giant cancer cells

**DOI:** 10.1017/erm.2025.10028

**Published:** 2025-12-10

**Authors:** Lakshmi Vineela Nalla, Siva Nageswara Rao Gajula

**Affiliations:** 1 Department of Pharmacology, GITAM School of Pharmacy, GITAM (Deemed to be University), Visakhapatnam, India; 2 Department of Pharmaceutical Analysis, GITAM School of Pharmacy, GITAM (Deemed to be University), Visakhapatnam, India

**Keywords:** 3D culture, experimental models, organoids, polyploidy, tumour explants, xenografts

## Abstract

**Background:**

Polyploid Giant Cancer Cells (PGCCs) are a malformed subpopulation of tumor. They play a crucial role in metastasis, recurrence, and therapy resistance. However, the inconsistent model systems and a lack of standardization have hindered mechanistic understanding and clinical translation. This review highlights the pluralistic research for clinical application by methodically analyzing various model systems used in PGCC research to fill the gap in the literature.

**Methods:**

As of November 2025, scholarly literature gathered from Google Scholar, PubMed, and ScienceDirect focused on examining the development, characteristics, and functional involvement of PGCCs in cancer.

**Results:**

In vitro approaches, although limited in their physiological relevance, enable detailed mechanistic studies and facilitate the screening of drugs. Ex vivo tumor explants and organoids preserve patient-specific traits with translational potential, while in vivo models, such as Drosophila and mouse xenografts, provide insight into PGCC function in complex tissue environments. By mapping model capabilities against PGCC research priorities, we demonstrate that no single system comprehensively recapitulates PGCC biology, necessitating integrated, multi-model experimental strategies that we outline in this study. More specifically, integrating patient-derived organoids with lineage-traced xenografts and single-cell omics enables continuous tracking of PGCC development and functional diversity, facilitating mechanistic studies of metastasis, drug resistance, and identification of clinical biomarkers for patient stratification.

**Conclusion:**

Considering the current lack of PGCC-targeted therapies, the convergence of model modification and the development of single-cell and imaging capabilities indicates significant progress toward therapeutically relevant findings. The ongoing development of these models is thus crucial for translating PGCC biology into predictive diagnoses and effective treatment methods.

## Introduction

Recently, advancements in cancer therapy have emerged for reducing mortality, recurrence and improving the quality of life for cancer patients. Despite these, many patients experience a shift from an initial positive treatment response towards a disease recurrence and relapse. This challenge, referred to as acquired resistance to therapy, endures as a considerable obstacle to achieve successful and effective treatment (Refs. 1, 2). Additionally, the existence of resistant cancer stem cells, a dearth of prognostic biomarkers and developing drug resistance restrict the efficiency of existing treatment (Refs. 3, 4). Recent studies have brought attention to the importance of PGCCs in tumours that drive the spread of cancer by serving as a reservoir of cellular plasticity and influencing the outcomes of chemotherapy and immunotherapy. PGCCs are a distinct and well-recognized sub-population originating in the complex tumour landscape by endoreplication, cell fusion and failure of cytokinesis in response to various chemical and environmental stressors. They acquire the properties of cancer stem cells and are resistant to treatments (Ref. 5). They also enter a condition of reversible senescence, reawakening in the absence of therapeutic insults and divide by amitosis or neosis to create resistant progeny (Ref. 6). Additionally, molecular data suggest that PGCCs maintain a variety of adaptations that enable them to selectively withstand harsh environments and boost their secretory factors in order to alter the tumour niche. Consequently, identifying and understanding PGCCs biology may aid in reducing the resistance of cancers to therapies (Ref. 7).

Research on PGCCs is often hindered by inconsistent terminology, which refers to the cells by several names in the literature, such as osteoclast-like giant cells, poly-aneuploid cancer cells (PACCs), multinucleated giant cells (MNGCs), hyperdiploid cells, pleomorphic cancer cells and large cancer stem cells (Refs. 8, 9). In the field, this absence of standard terminology makes it difficult to communicate clearly and advance together. For greater clarity, we have addressed this by including a PGCC identification checklist in Table 1. In contrast to tumour-specific events, PGCCs occurrence across various cancer types in response to diverse insults, including hypoxia, chemotherapy, radiation, viral infection and mechanical pressure, indicates that their formation is a conserved adaptive (Ref. 10). Regardless of the trigger, these cells consistently exhibit polyploidy, enlarged size and altered functions, suggesting the activation of a core cellular survival programme involving disrupted cell cycle control and DNA damage response pathways (Ref. 11).

**Table 1.** Recommended checklist for PGCC identification

Terms in use (primarily by) PGCC: Polyploid giant cancer cell (cell biologists) ETC: Endopolyploidy tumour cells polyploid/polyploidizing/depolyploidizing (cell biologists) Giant MNGC: Multi-nucleated and giant cells (cell biologists or pathologists) Monster monstrocellular (pathologists) OGC: Osteoclast-like giant cells (pathologists) Pleomorphic anaplastic syncytial type (pathologists)			
Category	Criteria	Representative markers or methods	Inference
Morphological	Cell diameter >3× that of neighbouring diploid cancer cells Presence of multiple or enlarged nuclei Abundant cytoplasm with vacuolation	Phase-contrast microscopy H&E staining Nuclear staining (DAPI, Hoechst)	Essential baseline criterion for PGCC identification
Cytological	Polyploid or multinucleated morphology confirmed by DNA content analysis	Flow cytometry (>4 N DNA content) Confocal imaging Cytogenetic assays	Distinguishes true polyploidy from simple multinucleation
Molecular	Elevated expression of cell cycle stress related markers and polyploidy-associated genes	p53, p21, γ-H2AX, Aurora B, Ki–67, cyclin B1	Indicates checkpoint escape and DNA damage adaptation
Cytoskeletal/structural	Reorganization of actin or tubulin networks Increased nuclear–cytoplasmic ratio heterogeneity	Phalloidin staining Tubulin immunofluorescence	Reflects dynamic remodelling typical of PGCCs
Functional behaviour	Ability to generate progeny through asymmetric budding or neosis Reversible depolyploidization capability	Live-cell imaging Time-lapse microscopy	Confirms functional versatility beyond morphology
Stemness Features	Expression of pluripotency or EMT-associated markers	CD44, ALDH1, Nanog, OCT4, SOX2, Zeb1	Correlates with therapy resistance and tumour repopulation
Stress Induction Context	Typically induced by chemotherapy, hypoxia or radiation stress	Cisplatin, paclitaxel, hypoxia-mimetic or irradiation models	Establishes the stress-mediated origin of PGCCs

Large multinucleated cells have been observed in tumours for almost 150 years, with early reports by pioneers such as Johannes Muller and Rudolph Virchow (Ref. 12). However, during those times, PGCCs were mostly disregarded as either artefacts from tissue handling and culture or as senescent and non-dividing abnormalities. These inaccuracies resulted from the limitations of conventional research techniques, such as 2D cultures and short-term trials, which led to an inadequate ability to capture the whole spectrum of PGCC behaviour. Recent advances in imaging, molecular analysis and multi-model systems have altered this viewpoint, upholding PGCCs as a dynamic drivers of cancer biology (Ref. 13). This inaccuracy resulted from the limitations of conventional research techniques, such as 2D cultures and short-term trials, which were inadequate for capturing the whole spectrum of PGCC behaviour. This review process examines the current knowledge of PGCCs biological traits and their development. Additionally, it offers investigative evidence across various model platforms (*in* *vitro*, *ex* *vivo* and *in* *vivo*) to advance therapeutically driven research for targeting PGCC-driven disease progression.

## Methodology

As was highlighted, this review illustrates the model systems for the development of PGCCs. A literature search was conducted online using several databases and search engines, such as PubMed, Google Scholar and ScienceDirect, to explore this topic. As of November 2025, the most pertinent articles supporting this study were found using keywords such as ‘polyploid giant cancer cells’, ‘PGCCs’, ‘polyploidy’, ‘2D cultures’, ‘3D cultures’, ‘spheroids’, ‘organoids’, ‘*in* *vitro*’, ‘*ex* *vivo*’, ‘*in* *vivo*’, ‘xenograft’, ‘allograft’, ‘*Drosophila*’, ‘hypoxia’, and ‘*in silico*’. Furthermore, publications with mismatched or irrelevant keywords were excluded from the study analysis. Based on their applicability and suitability for the present topic of discussion, each article was reviewed and cited. To obtain pertinent search results, we utilized PubMed’s ‘Boolean Operators’ (AND, NOT and OR) to achieve this. The inclusion criteria include (1) studies that investigated specifically PGCC formation, characterization and explored functional relevance with clinical outcome, (2) the article must include well-established 2D and 3D *in* *vitro* models, ex vivo, in vivo or in silico models and (3) the article must provide mechanisms, clinical and translational relevance of PGCCs biology. Among the exclusion criteria are the articles that neither lack proper methodology nor are unrelated to cancer biology or unrelated polyploidy. The titles and abstracts were screened, and further, the full texts were evaluated for eligibility. We further evaluated studies using a scoring system of 0–3. The number of studies meeting each validation threshold is summarized as follows: 3 studies showed minimal validation (score ≥1), 18 studies showed intermediate evidence (score ≥2) and 10 studies achieved full functional validation (score 3). PGCCs are defined not only by their phenotypic identification but also require functional validation to confirm their identity. Validated PGCCs exhibit additional features, including the ability to generate progeny through asymmetric division, tumourigenicity in suitable *in* *vivo* models, expression of cancer stem cell markers and resistance to therapeutic agents. This distinction is critical for the accurate interpretation of data and assessment of therapeutic targeting strategies.

## Biological characteristics of PGCCs

**Figure 1.**
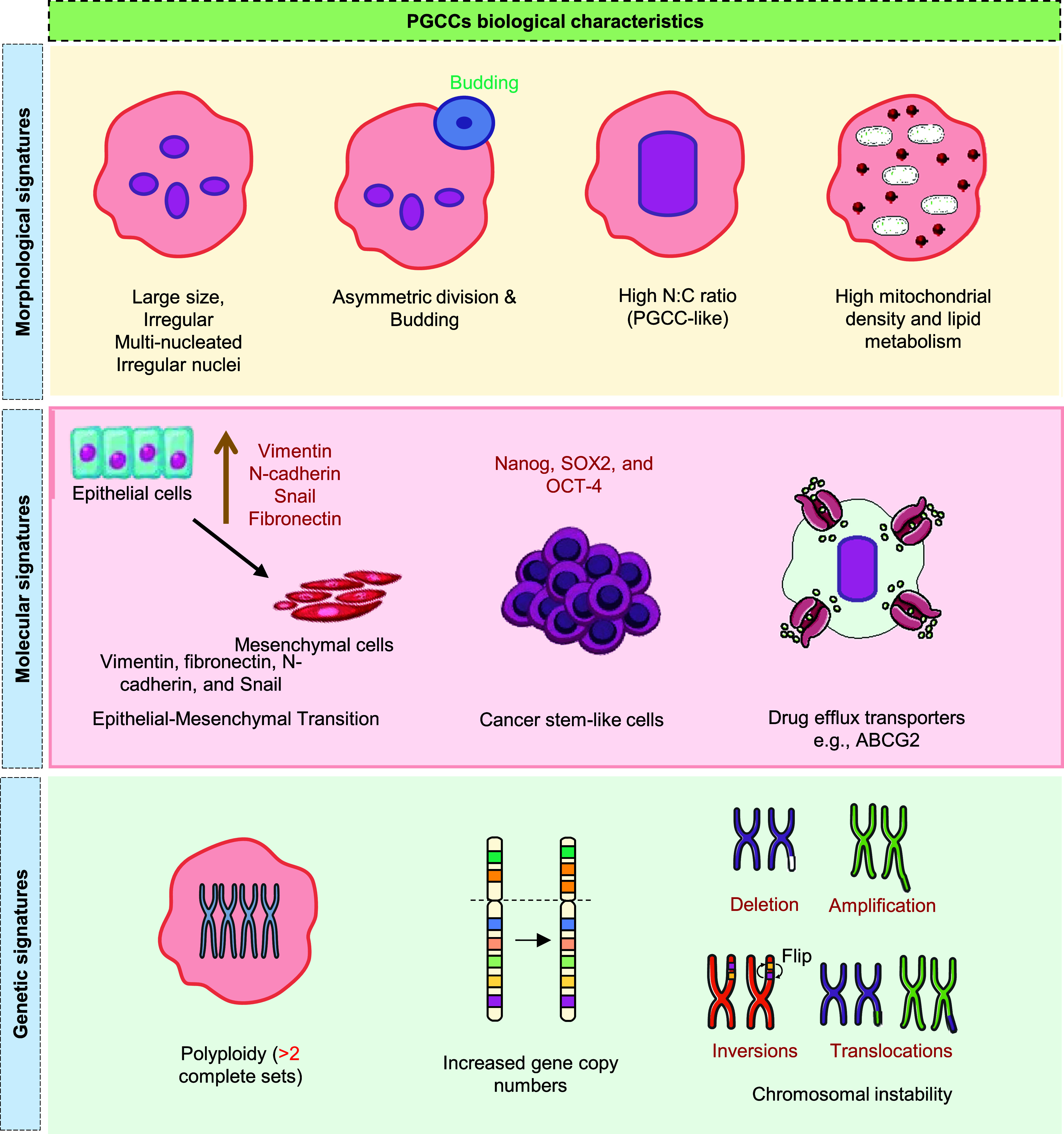
Key biological characteristics of PGCCs. Overview of key morphological (enlarged size, multinucleation, budding, high N:C ratio, mitochondrial enrichment), molecular (EMT markers, stemness factors, drug efflux transporters) and genetic (polyploidy, elevated gene copy numbers, chromosomal instability) signatures that define PGCC biology.

PGCCs represent a distinct subset of cancer cells, marked by their unusually large size and multiple nuclei. As illustrated in Figure 1, PGCCs exhibit distinct morphological, molecular and genetic signatures, including enlarged and multinucleated morphology, epithelial–mesenchymal transition (EMT) and stemness-associated markers and widespread genomic instability, often exhibiting heightened aggressiveness and increased resistance to therapy (Refs. 14, 15). Additionally, PGCCs exhibit distinct gene expression patterns associated with cell cycle regulation, DNA repair and drug resistance. White-Gilbertson et al. employed RNA sequencing to explore transcriptomic changes associated with the transition of cancer cells through polyploidization and subsequent depolyploidization. These findings highlighted CDKN1A/p21, a key cell cycle inhibitor, as a central regulator in the formation of PGCCs and their initial progeny. Further, PGCCs up-regulate p21 expression in the cytoplasm and blocking p21 expression using UC2288 suppresses PGCC formation and impairs the generation of progeny from PGCCs, improving therapeutic efficacy (Ref. 16). Contrary to this, inhibition of p21 increases the susceptibility of cancer cells to polyploidization (mitotic error), thereby increasing the sensitivity of cells to mitotic inhibitors (Ref. 17). In certain settings, PGCC can also emerge as a result of p53 mutation or deficiency, which states the p21-independent mechanism (Ref. 18). Therefore, the involvement of p21 is not universal and is highly context dependent for PGCC formation.

In addition, the most consistent expression of anti-apoptotic proteins such as Bcl-2, Bcl-xL, Survivin and Beclin were significantly elevated in multiple studies on PGCC across various model systems, explaining the enhanced resistance of PGCCs to apoptosis (Ref. 19). In general, the expression of anti-apoptotic proteins is often observed as an adaptive response to various forms of stress. Furthermore, it remains unclear whether these modifications serve as a compensatory mechanism or a triggering event in PGCCs. Ongoing temporal and mechanistic research is being done to comprehend these changes. Moreover, pharmacological inhibition with ABT-263 or the use of siRNAs against Bcl-xL, combined with ZM447439 (ZM), resulted in cooperative inhibition of drug-resistant polyploid cells in acute myeloid leukaemia (Ref. 20). This suggests that anti-apoptotic proteins play a crucial role as a key survival factor for PGCCs. Evidence on hypoxia-inducible factor (HIF)-1α’s involvement in PGCC formation has revealed that its subcellular distribution is influenced by SUMOylation at lysine sites K391 and K477. Moreover, increased nuclear localization of HIF-1α has been linked to promoting a more aggressive phenotype in the progeny of PGCCs (Ref. 21). Furthermore, whole-genome duplication (WGD) without subsequent mitotic division results in cellular enlargement, elevated nuclear genome ploidy and the formation of a large polyploid cell containing the complete set of genetic material (Ref. 22).

According to scientific studies, PGCCs frequently exhibit increased aggression, the capacity to spread and resistance to treatment. Research by Zhang and colleagues in prostate cancer indicates that larger, multinucleated cancer cells exhibited greater aggressiveness and metastatic potential than the original cells (Ref. 23). Their experiments involved injecting GFP-labelled PC-3 cells into mice, allowing metastasis to lymph nodes and re-injecting the metastasized cells. After six rounds of this selection process, the resulting PC-3-GFP-LN cell line showed significant enrichment of giant, often multinucleated (up to 22 nuclei) cells. This cell line displayed a strong ability to metastasize to various sites (lung, bone and lymph nodes) and demonstrated increased resistance to standard chemotherapy drugs like cisplatin, doxorubicin and 5-fluorouracil (Ref. 23). Similarly, Weihua et al. investigated spontaneously occurring multinucleated cells in the UV-2257 murine fibrosarcoma cell line using both in vitro and in vivo methods (Ref. 24). Live imaging revealed that multinucleation could arise from a failure of cell division and that giant cells could themselves divide to produce more giant cells. These larger cells were also found to be more resistant to doxorubicin. Notably, they possessed self-renewal capabilities and could grow independently of attachment, a hallmark of cancer. By physically separating giant cells using filters, the researchers demonstrated that even a single giant cell could initiate tumour growth and lung metastasis when implanted in mice (Ref. 24). In continuation, the connection between polyploidy and cancer is increasingly documented. For instance, Hasegawa et al. reported a link between multinucleated giant cancer cells and cancer-associated fibroblasts in the spread to peritoneal metastasis of mouse pancreatic cancer (Ref. 25). To conclude, multiple studies across various cancers have demonstrated that PGCCs with distinct biological characteristics induce EMT, a pivotal process in metastasis, cancer progression and the development of drug resistance. The extensive experimental and computational literature, encompassing various models for the development and characterization of PGCCs, is covered in the section next.

## Investigational models for PGCC study

Even though PGCCs are frequently uncommon, the idea that they are ‘keystone species’ in the tumour ecosystem emphasizes how important they are to the development of tumours. The elimination or disturbance of PGCCs may destabilize the entire tumour architecture, reducing its potential for growth, metastasis and resistance to treatment, much like keystone species in ecological systems (Ref. 6). This change in perspective emphasizes how ineffective it is to focus just on the bulk tumour population to achieve successful and curative results. Because of their scarcity, fleeting nature and significant functional importance, PGCCs require specialized and complex model systems for study. These models are essential for uncovering the molecular mechanisms driving PGCC formation under stress conditions like radiation, chemotherapy and hypoxia (Ref. 8). These models also enable detailed investigation of the distinct biological characteristics of PGCCs, including altered metabolism, neosis and stem cell-like properties, along with their dynamic interactions within TME, such as immune system evasion and stromal remodelling (Ref. 8). Moreover, experimental systems are crucial for designing and conducting pre-clinical evaluations of therapies targeting PGCCs and for validating their roles in cancer recurrence, metastasis and treatment resistance (Ref. 26). Therefore, the strategic development of diverse PGCC models is crucial for advancing our understanding of their biology and translating this knowledge into targeted cancer therapies. The following section provides an in-depth understanding of various models for PGCCs induction.

### *In vitro* models

Two-dimensional (2D) cell culture remains a fundamental tool in cancer research as it offers cost-effective and high-throughput screening, supporting advances in diagnosis, prognosis and therapy. While ongoing innovation in cancer treatment requires continuous refinement, the inherent variability of cancer underscores the need for personalized approaches. On the other hand, three-dimensional (3D) culture systems activate a range of autocrine, paracrine and cell-specific responses that closely mimic key elements of cancer progression observed *in* *vivo*, which are often not fully replicated in 2D models.

#### Two-dimensional cell culture systems

Two-dimensional (2D) monolayer cultures remain a cornerstone model in PGCC research, as they offer a flexible and adaptable platform for method development and skill enhancement. In addition, it is considered one of the most cost-effective, efficient and sustainable techniques available to both researchers and clinicians. A variety of stressors can induce the transition of cancer cells into the PGCCs. Despite their many benefits, they also have drawbacks. In 2D culture systems, rigid substrates force unnatural cell spreading, which distorts their morphology, affects polarity and signalling. It also imposes artificial constraints on cell shape compared to 3D cultures. Furthermore, 2D cultures exacerbate stress responses by triggering the production of reactive oxygen species and altering cellular stress signalling (Ref. 27). Crucially, 2D cultures highlight the inadequacy of capturing cellular heterogeneity and cell fate plasticity, which are more effectively achieved with 3D systems (Ref. 28). Therefore, it is recommended to validate the 2D findings with orthogonal techniques such as flow cytometry and live cell lineage tracing in 3D systems for morphology and polyploidy assessments to better recapitulate cell–cell and cell–matrix interactions (Ref. 29). Although 2D cultures are used due to their simplicity, they often fail to accurately represent the cell growth process observed in the physiological milieu *in* *vivo*. The lack of a complex and biologically rich environment in these 2D systems might be the source of this difference.

**Chemotherapeutic agents.** A varied range of cytotoxic drugs are a potent inducer of PGCCs via distinct pathways, resulting in different PGCC traits and functional consequences. Antimitotic agents such as paclitaxel (PTX) and docetaxel stabilize microtubules and frequently cause mitotic arrest followed by mitotic slippage, that is, without proper chromosome segregation or cytokinesis. This process allows polyploidization, often leading to senescence and cancer stemness (Refs. 30–32). The PGCCs that arise from this mechanism generally exhibit enhanced lineage flexibility and produce offspring via an amitotic process. Clinical data support this, since a high PGCC count was associated with a poor outcome in 51 laryngeal cancer patients clinical tissue, indicating a link between pre-clinical and clinical findings (Ref. 33). In parallel, DNA-damaging agents such as cisplatin, carboplatin and doxorubicin can induce DNA replication without cell division via endoreplication or endocycling, triggering cell cycle arrest mechanisms that result in PGCC formation (Refs. 34–36). PGCCs generated by this process frequently display therapy-induced senescence characteristics, including an altered senescence-associated secretory phenotype (SASP) that is linked to tumour growth and therapy resistance. Research evidence with other drugs, such as arsenic trioxide (ATO) (Ref. 37), staurosporine (Ref. 38) and gemcitabine (Ref. 39), has also been reported to induce PGCCs. Nevertheless, their induction of polyploidization in cancer cells is due to higher apoptotic stress, mitochondrial malfunction or nucleotide pool imbalances. Notably, higher drug concentrations result in a larger percentage of PGCCs, indicating the induction is frequently dose dependent. Since PGCCs caused by mitotic slippage may differ significantly from those generated by endoreplication in terms of proliferative capacity, adaptability and therapeutic resistance, it is essential to understand these mechanistic differences. Therefore, the selection of model systems and translational approaches targeted at these robust tumour cell types can be better informed by the molecular categorization of drug-induced PGCCs.

**Hypoxia.** Hypoxia is a known stressor essential for the development and survival of PGCCs in the tumour microenvironment. One crucial physiological cause for developing PGCC is the low oxygen tension in many solid tumours. This can be duplicated experimentally using chemical hypoxia mimetics, such as cobalt chloride (CoCl_2_) (Ref. 35), self-generated hypoxia models (Ref. 36) or cultivating cells in hypoxic chambers with an oxygen concentration of 0.1% (Ref. 40). According to phosphorescence-based oxygen sensing, metastatic prostate cancer cells produce their hypoxic zones, where cell size intricately affects both cellular motility and survival (Ref. 41). This has been linked to activating essential signalling pathways, such as p38 MAPK, ERK, JNK and CDC25C, indicating a complex molecular framework through which hypoxia facilitates cellular reprogramming and polyploidization in colon cancer (Ref. 42). Notably, CoCl_2_ therapy frequently enriches the more resilient distributed PGCC population with a branching appearance while selectively killing diploid cancer cells (Table 2) (Ref. 43). Mechanistically, stabilization of HIF-1α by CoCl_2_ occurs via iron chelation. It induces hypoxia that differs from true low-oxygen environments by bypassing the canonical oxygen-sensing machinery, resulting in a redox imbalance, varied metabolic flux and cellular responses. Literature evidence from global gene expression analysis revealed differential gene expression between the two models, with the effect of hypoxia induction being time sensitive across models (Ref. 44). It also exhibits distinct regulatory mechanisms at transcriptional and post-transcriptional levels, independent of HIF stabilization (Ref. 45). Therefore, these conclusions should be considered before model selection, as they will have profound implications for the interpretation of studies using chemical hypoxia. These results highlight hypoxia as a key factor in the development of PGCC and its role in shaping the tumour’s aggressive and adaptable characteristics. Further, an integrated understanding highlights the careful model selection to explore the PGCC biology under hypoxic conditions, driving cancer progression.

**Table 2.** Summary of various studies detailing the model development, scoring system, isolation methods, molecular markers and functional characterization of PGCCs across different cancer types

Cancer type	Cell lines used	Model development	Scoring system (0–3)	Isolation techniques	Polyploidy verification method	Molecular markers evaluated	Functional assays performed	Chromosomal/CLN analysis	Daughter cell generation	References
Prostate cancer	PPC1, U118MG and HT29 cells	Single dose of gamma irradiation (137Cs γ-irradiator), 5 nM docetaxel or 10 μM cisplatin	2	Size exclusion filtration using a 20-μ cell strainer	Flow cytometry (increase in % cells with >4n genome)	p21, p53, Histone H3	Colony-forming assay, Western blotting and RNA-seq analysis	✖	✓ (Asymmetric division)	(Ref. 16)
	PC3 cells	Cisplatin (6 μM)	2	PluriStrainer^Ⓡ^ 15 μm cell strainer	Nuclear morphological analysis (increased in nuclear volume), DAPI intensity	Annexin V, lamin A/C	Click-It Plus, dU assay, Cell cycle analysis	✖	✖	(Ref. 36)
	PPC1, U118MG and HT29 cells	137Cs γ-irradiator	2	20 μ filter (Pluriselect)	Flow cytometry (G2/M+ composed of polyploid cells)	ASAH1, S1P	Lipidomic analysis, colony formation assay	✖	✓ (Non-mitotic primitive cleavage-like fashion)	(Ref. 94)
Glioma	U251 (high-grade) and Hs683 (low-grade) cells	Lentiviruses infection (PHA-DMSO-PEG fusion method)	1	Puromycin screening and flow cytometry	Cell size and DNA content	-	Proliferation assay	✓	✖	(Ref. 95)
	HCT 116, SF–539 and SNB–19	ST–401, inhibitor of microtubule (MT) assembly	2	Stress-induced selection	Flow cytometry using DAPI	eIF2α, p53, tubulin, pericentrin, G3BP1 and γH2AX	Apoptosis, cell cycle analysis, autophagy, scRNAseq analysis and mitochondrial function assays	✓	✖	(Ref. 96)
	A172 cells	100 μM CoCl_2_	2	Treatment and recovery	Morphological characterization	Nestin, HIF–1α, CD133, OCT–4	Single-cell cloning, tumoursphere formation assays, RT-PCR, western blotting	✖	✓ (Asymmetric division called neosis)	(Ref. 97)
Breast cancer	MCF7, MDA-MB–157, MDA-MB–231, T47D, HCC1806 and BT549	DMSO or FiVe1	3	Fluorescence-activated cell sorting	Multi nucleation and FACS analysis for DNA content	Vimentin, Integrin β4, E-Cadherin, cytokeratin 18, cytokeratin 8, Numb and FOXC2	Mammosphere assay, Western blot analysis, Soft agar and tumourigenesis assays, Matrigel invasion assays, Stem cell division assay, metastatic studies in vivo	✖	✓ (Symmetrical stem cell division)	(Ref. 98)
	MDA-MB–231, MDA-MB–436, MDA-MB–468, Vari068 and BT474 cells	1 μM Docetaxel treatment	2	Flow-sorted	Live/Dead/Hoechst staining (top 1% Hoechst high for PGCCs and bottom 5% Hoechst low for non-PGCCs)	FTL, FTH1,CCNB1, CDC20 and SLC3A2	G-banding metaphase analysis, Spheroid formation, drug testing on-chip, scRNA-Seq, real-time PCR	✓	✖	(Ref. 31)
	MDA-MB–231	Fludioxonil (10–5 M)	2	Flow cytometry	Live-cell image system (Hetero-, enlarged and multinucleated shape)	p53, Cyclin D1, Cyclin E1, Cyclin A1, ERK1/2, NF-κB	Cell cycle analysis, WST assay, DAPI and actin staining, cell migration, next-generation sequencing (NGS)	✖	✓ (Amitotic and budding division)	(Ref. 50)
Breast and ovarian cancer	BT–549 and HEY cell lines	CoCl_2_ treatment	3	Treatment and recovery	Hematoxylin and eosin staining to measure the size of nuceli	Cyclin B1, CDC25B, CDC25C, Chk2 and Aurora-A kinase,	Immunocytochemistry, Western blotting, Cell migration and Invasion assays and in vivo (nude mice, s.c.)	✖	✓ (Asymmetric cell division)	(Ref. 99)
Nasopharyngeal cancer	CNE2, C666–1, TW03 EBV (−) and TW03 EBV (+)	Paclitaxel (PTX) 150 ng/μL	1	Chemotherapy-induced selection	Morphological changes	CD133, CD49f, CD44, ALDH1A1, CD24, SOX2	Immunocytochemistry, Cell cycle analysis	✖	✖	(Ref. 32)
	CNE–2	Paclitaxel	3	Treatment and recovery	Immunofluorescence	Autophagy markers	Live/Dead cell double staining assays, RNA-seq, mouse recurrence model	✖	✓ (Burst-like cell divisions)	(Ref. 100)
	5–8F and CNE–2	150 ng/μL paclitaxel	3	Treatment and recovery	PI-FACS analysis	Cyclin E, cyclin B1, cyclin D1 and CDK8	Morphologic characteristics, Mitochondrial morphology, Autophagy, β-Gal staining, in vivo (nude mice)	✖	✓ (Burst-like cell divisions)	(Ref. 101)
Lung cancer	Phase II clinical trial patients of squamous-cell carcinoma (38%) or adenocarcinoma (59%)	Chemoradiation and Atezolizumab	2	CellSieve system	Multiplex immunostaining (CAMLs ≥50 μm)	CD45/CD14/CD11c	Circulating multinucleated myeloid cells ≥50 μm	✖	✖	(Ref. 102)
	A549 and NCI-H1299	Docetaxel at 100 nM, 24 h	3	Treatment and recovery	DNA content of cells was analysed by flow cytometry (DNA > 4 N)	Cdc2, p-Cdc2, RB, p-RB, E2F1, p53 and p21	Cell cycle and DNA content analysis SA-β-Gal staining, xenograft tumour model	✖	✖	(Ref. 68)
Drosophila solid tumour	Actts-Gal4 and Mmp1ts-Gal4		3	40-μm filter	DAPI intensity analyses, flow cytometer	MMP1 and JNK	BrdU labelling, Live imaging, Flow cytometry	✓	✓ (Continued endoreplication)	(Ref. 64)
Colon cancer	HCT–116 cells	Doxorubicin (200 nM)	1	Treatment and recovery	Flow cytometry and microscopy using DAPI	High activity of β-galactosidase	Microscopy, Flow cytometry	✖	✖	(Ref. 103)
	LoVo and HCT116 cell lines	CoCl_2_ (450 μM)	3	Stress-induced selection	Three times larger in size, Scattered morphology Nucleus measurement using a micrometer	S100A4, cathepsin B, cyclin B1, TRIM21 and Annexin A2	Cell migration and invasion assay, Plate Colony Formation assay, Western Blot, immunocytochemistry, BALB/cNU/NU nude mice	✖	✓ (Budding)	(Ref. 42)
	LoVo and HCT116	Neoadjuvant Chemoradiation (Capecitabine, oxaliplatin, irinotecan and with irradiation, 9 Gy at a dose of 1.0 Gy/min)	2	Stress-induced selection	Three times larger in size, Scattered morphology Nucleus measurement using a micrometer	N-cadherin, Vimentin, E-cadherin, Fibronectin, Snail and Slug, Twist–1 and CK7	Cancer stemness	✖	✓ (Asymmetric cell division)	(Ref. 47)
Prostate cancer	Human prostate epithelial cells (PECs)	Human cytomegalovirus infection	2	–	Microscopy and flow cytometry using PI (Increased polyploidy)	IE–1, Myc, EZH2, Nestin, Nanog, SOX2, Vimentin and E-cadherin	Soft agar colony formation assay, Spheroid formation assay	✖	✖	(Ref. 104)
Prostate and Lung cancer	PPC1, A549, SW620	Docetaxel (5 nM), 137Cs γ-irradiator (8 Gy), cisplatin (30 μM)	2	Flow cytometry, 20-micron filter	Flow cytometry (>2 full copies of genetic material)	CD133, ASAH1, DES1, SK1	Morphological changes, ALDH assay, Sphingolipids	✖	✓ (Asymmetric cell division by neosis)	(Ref. 105)
Liver cancer	SK-hep1 and PLC/PRF/5 cell lines	Mycoplasma hyorhini infection	3	–	Flow cytometry (High smFISH signal intensity)	MFN 1, CD133, CD44, EpCAM	Time series imaging, H&E staining, Immunohistochemistry Western blot, Analysis of mitochondrial morphology, Proteomics, Metabolomics, BALB/c nude mice	✖	✓ (Endoreduplication)	(Ref. 106)
Ovarian cancer	HEY cell line	450 μM of CoCl_2,_ 48 hrs	2	Stress-induced selection	–	Cyclin E, SKP2	Tissue samples, Immunocytochemical analysis, Western blot analysis	✖	✓ (Budding)	(Ref. 107)
	HEY and BT–549 cells	450 μM CoCl_2_ for 48 and 24 h respectively	2	Stress-induced selection	–	p38MAPK, ERK, JNK and CDC25C	Immunocytochemical analysis, Western blot analysis	✖	✓ (Budding)	(Ref. 43)
	SKOV3 and A2780	Docetaxel (800 nM), overnight	2	Treatment and recovery	Flow cytometry analysis (DNA > 4 N was increased)	γ-H2A.X, KLF4, OCT4	Cell cycle and DNA content analysis, β-galactosidase assay, Mitochondrial membrane potential (Ψm)	✖	✖	(Ref. 30)
	Hey, SKOV3, OVCA–432, OVCAR8, OVCAR5 and PEO–1	Olaparib	3	FACS	Flow cytometry using PI (DNA > 4 N was increased)	p21, GATA4	Western blotting, Microscopy, Time-lapse tracking, Highly resistance, SA-β-gal staining, Athymic nude (nu/nu) mice xenograft study	✓	✓ (Multipolar mitosis and restitution multipolar endomitosis)	(Ref. 56)
	HOSE cells or OECs	Human cytomegalovirus infection	2	–	Flow cytometry using PI (≥4 N) and tetraploid cells (4 N)	IE1, pp65, Myc, EZH2, Sox2, Nanog, E-cadherin and vimentin	Telomerase activity, Cell cycle analysis, Colony formation assay	✖	✖	(Ref. 108)
Cervical cancer	C33a and HeLa cells	6 mV X-ray source (7 Gy or 14 Gy)	2	–	DNA ploidy analysis	LC3-I, LC3-II, p62	MDC and Lyso tracker red staining, Mitochondrial membrane potential (MMP)	✖	✖	(Ref. 109)
Lymphoma and cervical cancer	Namalwa Burkitt’s lymphoma cells, HeLa S3 cells	Irradiation (10 Gy dose)	2	–	DNA image cytometry	Aurora-B, Lamin B, p53	Cell cycle analysis, LSDCAS Imaging Analysis, Immunofluorescent staining	✓	✓	(Ref. 48)
Renal and breast cancer	HEY, HEK293T and MDA-MB–231	450 μM CoCl_2_ BALB/c nude mice	3	Stress-induced selection	Flow cytometry	OPN, SOX2, CD133, CD44	Alcian blue staining, Immunocytochemistry, Animal xenograft model	✖	✓ (Asymmetric cell division by neosis)	(Ref. 110)
Oral cancer	Cal33 and FaDu	Non-apoptotic doses of cisplatin 15 μM	2	Treatment and recovery	–	SIRT1, DNM1L and MFN1	Enhanced CASP3/7 activity, Antioxidant enzymes, Morphological changes	✖	✓ (Budding)	(Ref. 111)

*Note.*

1. Scoring system (0–3): Score 0 indicates identification based solely on morphological phenotype without functional validation. Score 1 denotes minimal validation, with confirmation of polyploidy by DNA content or nuclear size. Score 2 reflects intermediate validation, including functional evidence such as progeny generation or expression of cancer stem cell markers. Score 3 represents full validation, demonstrating tumourigenicity in vivo, ability to generate progeny cells through asymmetric division and molecular characterization confirming bonafide PGCC identity.

2. Morphological identification through 2D models is prone to artefacts and should be interpreted cautiously.

Abbreviations: DAPI: 4′,6-diamidino-2-phenylindole; ASAH1: N-acylsphingosine amidohydrolase 1; S1P: sphingosine-1-phosphate; G3BP1: Ras GTPase-activating protein-binding protein 1; γH2AX: phosphorylated histone H2AX; eIF2α: eukaryotic initiation factor 2 alpha subunit; HIF-1α: hypoxia-inducible factor 1, alpha subunit; CD133: prominin-1; OCT-4: octamer-binding transcription factor 4; FACS: fluorescence-activated cell sorting; FOXC2: forkhead box C2; FTL: ferritin light chain; FTH1: ferritin heavy chain 1; CCNB1: cyclin B1; CDC20: cell division cycle 20; SLC3A2: solute carrier family 3 member 2; ERK1/2: extracellular signal-regulated kinases 1 and 2; NF-κB: nuclear factor kappa-light-chain-enhancer of activated B cells; CDC25B: cell division cycle 25B; CDC25C: cell division cycle 25; Chk2: checkpoint kinase 2; ALDH1A1: aldehyde dehydrogenase 1 family member A1; CD24: cluster of differentiation 24; SOX2: sex determining region Y-box 2; CDK8: cyclin-dependent kinase 8; Cdc2: cell division control protein 2; RB (or pRB): retinoblastoma protein; E2F1: E2 promoter binding factor 1; p21 (or p21WAF1/CIP1): cyclin-dependent kinase inhibitor 1 (CDKN1A); p53 (or TP53): tumour protein p53; SA-β-Gal: senescence-associated β-galactosidase; MMP1: matrix metalloproteinase-1; JAK: c-Jun N-terminal kinase; S100A4: S100 calcium-binding protein A4; TRIM21: tripartite motif-containing protein 21; CK7: cytokeratin 7; EZH2: enhancer of zeste 2 polycomb repressive complex 2 subunit; DES1: dihydroceramide desaturase 1; KLF4: Krüppel-like factor 4; GATA4: GATA binding protein 4; pp65: phosphoprotein 65; IE1: immediate–early 1 protein; LC3-I: microtubule-associated protein 1 light chain 3-I; LC3-II: LC3 conjugated to phosphatidylethanolamine; p62: SQSTM1 (Sequestosome 1); LSDCAS: large-scale digital cell analysis system; OPN: osteopontin; smFISH: single-molecule fluorescence in situ hybridization.

**Irradiation.** Induction of PGCCs is primarily dependent on radiation, especially when therapy-induced stress responses are present. Ionizing radiation exposure can cause mitotic failure and DNA damage, which can cause cancer cells to become polyploid. Instead of solely resulting from cellular damage, these irradiation induced PGCCs can survive, endure and actively contribute to the growth of tumours (Ref. 46). Interestingly, it has been demonstrated that these PGCCs can re-populate tumours by a special mechanism known as neosis, in which they produce viable offspring with a renewed potential for proliferative activity (Ref. 46). The development of PGCCs after neoadjuvant chemoradiation in locally advanced rectal cancer has been linked to prognostic importance, indicating that they may serve as treatment response biomarkers (Ref. 47). Furthermore, endopolyploidy is associated with ongoing cell division activity in irradiation p53-deficient tumour cell lines, as shown by the expression of mitotic markers such as Aurora-B kinase (Ref. 48). These results emphasize the involvement of PGCCs in tumour persistence, recurrence and treatment resistance while highlighting their complex and adaptable character in response to irradiation.

#### Alternative stressors

The genesis of PGCCs in cancer is multifaceted, as evidenced by a range of different cellular stresses that drive their development. Multiple factors may contribute to PGCCs, including nutritional deficiency, chemicals, viral oncogenesis, pharmacological medications and mitotic stress, which may impact the proliferation of these highly adaptable cells. For example, glucose deprivation has been found to cause entosis, which might aid in developing PGCC as a survival mechanism in response to metabolic stress (Ref. 49). Research evidence observed that exposure to fludioxonil (10^−5^ M) for 72 h significantly reduced cell viability in MDA-MB-231 triple-negative breast cancer (TNBC) cells harbouring mutant p53 (mutp53), leading to the formation of PGCCs, characterized by enlarged cell bodies and an increased number of nuclei and increased the stemness and metastatic capacity (Ref. 50). In another study, bufalin was found to trigger the formation of PGCCs in LoVo and Hct116 cell lines. These PGCCs and their resulting progeny exhibited elevated expression of polo-like kinase 4 (PLK4). Moreover, the progeny cells demonstrated enhanced migratory and invasive properties, marked by the expression of proteins associated with EMT (Ref. 51). Besides, Herbein et al. demonstrated that the high-risk oncogenic strains of HCMV, characterized by increased EZH2 and Myc expression, can induce the formation of PGCCs, promote epithelial cell dedifferentiation and trigger the development of stem-like properties along with epithelial–mesenchymal and mesenchymal–epithelial transition (EMT/MET) traits, all occurring in conjunction with giant cell cycling. 2D cultures, while useful for preliminary PGCC investigations, fail to replicate the physiological relevance necessary to completely understand PGCCs’ functions in tumour development and therapeutic response due to the lack of complexity of the 3D tumour microenvironment.

#### Advanced 3D models

Three-dimensional cell culture methods have become the preferred approach for utilizing cancer cell lines to better connect purely *in* *vitro* studies with actual *in* *vivo* conditions. These systems have significantly advanced the study of various aspects of cancer biology, including cellular morphology, tumour microenvironment, gene and protein expression, invasion, migration, metastasis, angiogenesis, tumour metabolism, drug discovery, chemotherapeutic testing, adaptive responses and the behaviour of cancer stem cells. To overcome the limitations of 2D models, advanced 3D culture models have been developed.

**Spheroids.** Spheroid-based investigations are affordable, scalable and effective for high-throughput drug screening; however, they exhibit poor long-term stability and lack true tissue heterogeneity. Although hypoxic gradients do exist, they could be overstated in comparison to the real tumour microenvironments. Spheroids, particularly multicellular tumour spheroids (MCTS), mimic avascular tumour regions with hypoxic cores and are widely used to study PGCC formation under stress conditions like mechanical confinement. These models also support investigations into PGCC stemness, drug penetration and tumour initiation. Breast cancer spheroids formed in microfluidic non-adherent microwells resisted Docetaxel treatment, which was effective in 2D cultures, due to enhanced PGCC population. Subsequent 2-day treatment with anti-PGCC compounds Carfilzomib, ML162 or Thiostrepton eradicated drug-resistant population (Ref. 31) highlight that microfluidic spheroids capture drug-resistant mechanisms involving PGCCs. Furthermore, ARID1A knockdown in Caco-2 colon cancer cells enhanced self-aggregation, increased spheroid size, elevated PGCC numbers and up-regulated VEGF secretion, indicating a more aggressive cancer phenotype demonstrated by fluorescence imaging and hanging drop assays (Ref. 52). Additionally, alginate–gelatin microspheres encapsulating OVCAR8 spheroids treated with 25-SING retained their structure, exhibited a high PGCC content (~40%) and demonstrated resistance to paclitaxel. These cells replenish aggressive daughter cells via amitotic budding, a specific PGCC trait, highlighting distinct chemoresistant populations in spheroids compared to control group (Ref. 53). Following genotoxic stress, CD44^+^/CD133^+^ A549 NSCLC cells formed dormant PGCCs that re-entered the cell cycle to generate therapy resistant, invasive clones, suggesting PGCCs as a potential CSC source influenced by p53 status (Ref. 54). This demonstrates that checking the functional p53 is crucial for selecting therapeutic schemes for NSCLC patients.

**Figure 2.**
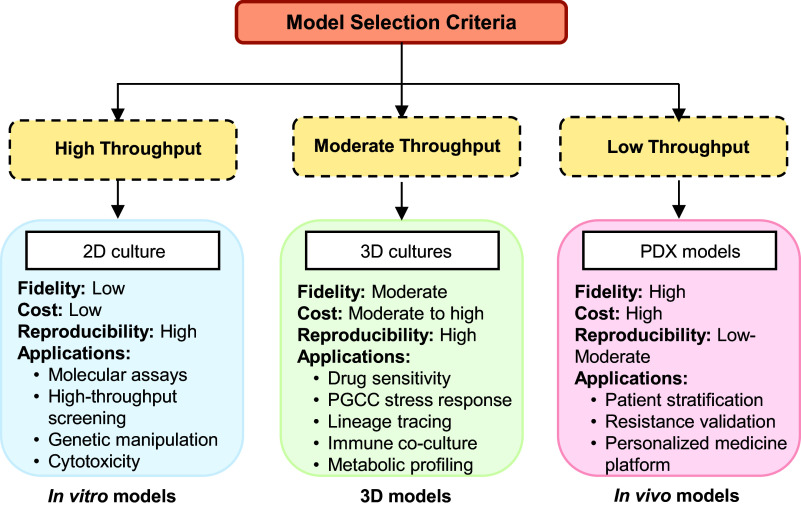
Decision-making framework. Overview of high-, moderate- and low-throughput experimental systems. 2D cultures provide low fidelity and high reproducibility; 3D cultures offer moderate-to-high fidelity with diverse functional applications; PDX models deliver high fidelity but with low throughput and higher cost.

**Organoids.** Cancer organoids or tumouroids, are advanced 3D models derived from pluripotent or adult stem cells or patient tumour tissues. Unlike cell line-derived spheroids, organoids retain architectural fidelity, genetic heterogeneity and better mimic the stem/progenitor dynamics of PGCCs. However, organoid culture is labour intensive, has lower throughput and exhibits high variability between patient samples. Patient-derived HGSC organoids cultured in Matrigel revealed dynamic tumour evolution driven by cyclic transitions between polyploid PGCCs and diploid cells, enabling malignant progression through spatio-temporal macro- and micro-evolution under stress conditions (Ref. 55). Furthermore, sub-lethal olaparib treatment significantly increased PGCC formation in HGSC-derived organoids and patient-derived xenografts, particularly in Org-3008, Org-2445 and Org-2414. These olaparib-induced PGCCs displayed senescence-like phenotypes, contributing to PARP inhibitor resistance (Ref. 56). However, current organoid models often lack functional vasculature and comprehensive immune components, limiting their ability to replicate the in vivo TME fully. However, in cases where vasculature is important, this constraint may render organoid investigations for PGCC–immune interactions or therapeutic response meaningless. Thus, recent developments in vascularized organoids and immune-organoid co-cultures may provide physiological significance and therapeutic translation for PGCC biology, paving the way for future studies. To aid in understanding, we have incorporated a matrix that offers the reader helpful recommendations for model selection (Figure 2).

**Microfluidic devices.** Recent advances have leveraged microfluidic technologies to explore the biology and therapeutic resistance of PGCCs with high physiological relevance. A recent study employed microfluidic systems featuring three-dimensional microvascular networks to recreate the in vivo microenvironment, enabling examination of the extravasation behaviour of MDA-MB-231 breast cancer cells (Ref. 57). This work demonstrated that polyploid MDA-MB-231 cells exhibited markedly increased extravasation and enhanced cell–matrix adhesion, underscoring the utility of microfluidic platforms in modelling PGCC metastatic potential under realistic biophysical conditions. Another investigation utilized a microfluidic device to probe drug resistance mechanisms in TNBC. Through dynamic microfluidic culture, doxorubicin-resistant cells displaying enlarged phenotype and elevated genomic content, characteristics of PGCCs were generated (Ref. 58). This study linked the emergence of PGCC-like polyploidy with chemoresistance and epigenetic regulatory mechanisms, highlighting the value of microfluidics in dissecting tumour cell heterogeneity and therapeutic evasion. Furthermore, microfluidic prostate cancer-on-chip models have also been developed to co-culture prostate cancer cells with stromal cells, thereby recapitulating heterogeneous tumour microenvironments and allowing investigation of PGCC dynamics under drug gradients such as docetaxel (Ref. 59). This approach has facilitated the study of PGCC adaptation and survival in complex drug-exposure conditions.

Additional microfluidic strategies employing label-free and size-based cell sorting techniques using isosceles trapezoidal spiral microchannel have been applied to isolate large cancer cells, including PGCCs, from heterogeneous populations (Ref. 60). These platforms enable functional assays of extravasation, adhesion and metastatic potential, furthering understanding of PGCC biology in breast cancer and other malignancies. Collectively, these studies demonstrate that microfluidic technologies serve as powerful tools to model PGCC behaviour, drug resistance and metastasis in physiologically relevant contexts, thereby advancing mechanistic insights and therapeutic targeting of these clinically challenging cancer cell populations. Although these models offer excellent physiological relevance and control over microenvironment, they are limited in large-scale throughput drug screening, mechanical stresses, the need for specialized expertise and equipment and difficulties in isolating and accurately quantifying rare PGCC populations within heterogeneous cancer cell mixtures.

#### Co-culture models

Co-culture systems, including 2D and 3D spheroids and engineered scaffolds, investigate the interaction between cancer cells, including PGCCs, and the tumour microenvironment (TME), examining juxtacrine and paracrine signalling. These models help understand how stromal cells influence PGCC behaviour, such as promoting invasion or modulating immune responses, and allow the study of cell fusion events that may contribute to PGCC formation or dormancy. In a co-culture system, PGCC supernatant induced morphological changes and fibroblast reprogramming, as well as IL-6. Still, only paclitaxel treatment promoted polyploidy in fibroblasts, highlighting the role of IL-6 in drug resistance through PGCC formation and the reprogramming of fibroblasts (Ref. 61). In a study, culturing PGCC-derived daughter cells (PDCs) in an osteo/chondrogenic medium revealed that the CTCF/p300 axis enhances their differentiation by increasing RUNX2 acetylation, a key factor for its transcriptional activity and stability, highlighting the potential of targeting PDCs with RUNX2 agonists as a therapeutic strategy. Furthermore, Wu et al. demonstrated that lidocaine (1.5 mM) re-programmes CD8^+^ tumour-infiltrating immune cells by inhibiting PD-1, thereby enhancing their cytotoxic function and triggering immunogenic cell death in PGCCs (Ref. 62).

In vitro models offer valuable tools for studying PGCCs due to their controllability, cost-effectiveness and suitability for high-throughput screening. They enable precise manipulation of experimental conditions and reduce reliance on animal models. Advanced 3D systems, such as organoids and co-cultures, enhance physiological relevance by mimicking the tumour microenvironment better. However, in vitro models lack key in vivo features, such as vascularization, immune system complexity and systemic interactions, which limits their predictive power. Challenges specific to PGCC research include their rarity, transient nature and the insensitivity of standard assays to detect PGCC-specific responses. Therefore, while 2D and 3D models are essential for initial studies, findings must be validated in more complex biological systems.

### *In vivo* models

In vivo models are indispensable for investigating the behaviour of PGCCs within the complex physiological context of a living organism, including interactions with the host immune system, vasculature and distant organs during metastasis. These models allow for the assessment of systemic effects and long-term outcomes that cannot be fully replicated in vitro.

#### Drosophila models

*Drosophila melanogaster* is a valuable model for studying PGCCs, offering genetic tractability for mechanistic dissection, in vivo imaging capabilities and cost-effectiveness for screening models. Furthermore, research from *Drosophila* models also provides mechanistic insights that may be applicable to human cancers. The Notch signalling system is a highly evolved mechanism in humans and *Drosophila* that regulates cell fate, proliferation and differentiation. This signalling plays an important role in the mitotic-to-endocycle transition, increasing polyploidy by up-regulating cell cycle regulators. These regulators help cyclins degrade, causing the cell cycle to stop without division. This signal is also largely conserved in mammals; hence, the *Drosophila* model is used to investigate conserved Notch-mediated endoreplication (Ref. 63). In Notch-driven solid tumours, polyploidization via endoreplication is an early event, mirroring PGCC formation in human cancers (Ref. 64). Unscheduled endocycles produce PGCC-like cells that resist death, undergo reversible senescence, secrete pro-proliferative cytokines and generate aneuploid progeny, contributing to tumour progression (Ref. 65). Although informative, this model outlines the bounds of this regulatory mechanism’s use in human tumours. Further, this also acts as a powerful model for studying tumourigenesis in non-proliferative and damage-stressed tissues. The prostate-like accessory gland reveals how oncogenic signalling drives hypertrophy and pro-tumourigenic programmes in terminally differentiated, polyploid cells, paralleling features of human prostate cancer (Ref. 66). The wing disc model shows how unscheduled endocycling generates polyploid, apoptosis-resistant cells that trigger chronic Src-JNK-mediated wounding responses, reshaping tissue and mimicking tumour-promoting microenvironments (Ref. 67). Together, these systems illuminate conserved mechanisms linking polyploidy, stress signalling and tumour progression, underscoring *Drosophila*’s value for dissecting cancer biology and identifying therapeutic targets. These findings emphasize the integration of mammalian systems for PGCC biology elucidation.

#### Xenograft models

Xenograft models provide a physiologically relevant in vivo system for studying PGCCs formation, survival and therapeutic response. By implanting human cancer cells or patient-derived tumours into immunocompromised mice, these models closely mimic the tumour microenvironment and reveal the role of PGCCs in progression, resistance and relapse. They provide key insights into PGCC-driven tumour heterogeneity, dormancy and regeneration.

**Patient-derived xenografts (PDX).** PDX models are generated by directly implanting cells/fresh tumour tissue from a patient into immunodeficient mice (e.g. NSG™ mice). A significant advantage of PDX models is their ability to better preserve the histological architecture, cellular heterogeneity (including potentially rare PGCC populations), genetic characteristics and TME components of the original patient tumour compared to CDX models. In a study targeting PGCCs, mifepristone was found to suppress tumour growth in both olaparib-naive and olaparib-resistant HGSC patient-derived xenografts (PDX) models, indicating that while the combination of olaparib and mifepristone may benefit naïve tumours, mifepristone alone could be effective against resistant tumours (Ref. 56).

**Cell line-derived xenografts (CDX).** These traditional models involve implanting established human cancer cell lines (often subcutaneously, sometimes orthotopically) into immunodeficient mice. The A549 xenograft model showed that docetaxel (Doc) induced senescence, increased IL-1β expression and promoted PGCC formation in vivo, with IL-1β playing a role in docetaxel resistance by regulating PGCC development in non-small cell lung cancer (Ref. 68).

#### Genetically engineered mouse models

GEMMs (genetically engineered mouse models) are developed by introducing specific genetic alterations, such as oncogene activation or tumour suppressor deletion, often tissue specific, using systems like Cre-Lox. Unlike xenograft models that require immunodeficient mice, GEMMs allow tumours to develop spontaneously within an intact immune system, enabling the study of tumour–immune interactions and the full course of tumourigenesis. While the referenced studies focus more on established GEMMs than their creation for PGCC research, models like the Hi-Myc prostate cancer GEMM have been used to track PGCC formation during tumour progression (Ref. 69). GEMMs have also helped uncover signalling pathways such as c-Src/FOXM1 in breast cancer, where genetic alterations led to polyploidy as a notable phenotype (Ref. 70).

### *Ex vivo* models

To better translate findings from controlled laboratory models to human cancer, studying patient-derived materials is essential. These models provide a context for comprehending tumour biology that is more clinically relevant. ex vivo cultures of patient tumours help research uncommon and dynamic populations like PGCCs because they preserve features of the original tumour microenvironment, such as heterogeneity and cellular architecture (Ref. 56). In addition, analysing circulating tumour cells (CTCs) provides insights into real-time tumour growth and the potential for metastasis (Ref. 71). Exploring the presence, behaviour and clinical relevance of PGCCs in human cancers necessitates using diverse patient-derived models.

#### Primary tumour-derived cultures

These models represent biological conditions more than current cell lines, utilizing fresh patient tumour material. This approach cultivates isolated cells in vitro by breaking down fresh tumour biopsies or PDX tissue. By minimizing stromal components, specialized systems like the primary cancer culture system (PCCS) can enhance PGCCs or their precursors while also enriching malignant cells, including cancer stem-like cells (CSCs) (Ref. 72). The ability of PGCCs to generate progeny ex vivo and their viability have been confirmed through their successful identification and isolation from primary ovarian cancer cultures (Ref. 34). Functional and molecular studies of PGCCs obtained from patients are made possible by these cultures.

#### Tumour explants

Tumour explant cultures preserve natural architecture, cellular heterogeneity and tumour microenvironment (TME) elements, such as immune and stromal cells, by keeping tiny pieces of patient tumour tissue in vitro (Ref. 73). This physiologically relevant model allows the study of PGCCs in their natural niche and their responses to therapies. Variants like precision-cut tissue slices and agitation based systems enhance nutrient exchange and tissue longevity, making them useful for pre-clinical drug testing and evaluating immunotherapy responses (Ref. 74). However, limitations include short tissue viability, gradual loss of TME integrity and difficulty modelling long-term processes like metastasis (Ref. 74). These ex vivo models represent valuable intermediate systems. They offer higher patient relevance than cell lines by using primary tissue and, in the case of explants, preserving some TME context. This makes them particularly useful for studying patient-derived PGCCs and their immediate responses to perturbation, while being more experimentally tractable and less resource intensive than full in vivo studies.

#### Circulating tumour cells

Circulating tumour cells (CTCs) are cancer cells that shed from primary or metastatic sites into the bloodstream and can serve as prognostic indicators in various cancers. Analysing CTCs enables real-time monitoring of disease progression, treatment response and metastatic potential. Studies, such as those examining CCR5 activation and endocytosis in tumour-derived cells from breast cancer patients, suggest that specific features of circulating cells like PGCCs may offer valuable insights into clinical outcomes (Ref. 75). Using a sensitive liquid biopsy method (ISET®) with cytopathological analysis, circulating PGCCs were detected in 20.18% of carcinoma patients, with a higher prevalence in metastatic cases. Their presence was significantly associated with poor overall survival over a 44.7-month follow-up, highlighting their potential as a prognostic marker and a target for future therapeutic strategies (Ref. 10).

### *In silico* study

Foremost, computational modelling techniques are crucial for understanding PGCC development and its complex behaviour, especially when combined with experimental data from flow cytometry, live-cell imaging and spatial transcriptomics (Ref. 76). Research evidence suggests single-cell RNA sequencing (scRNA-seq) enables detailed profiling of individual cells, making it a powerful tool for identifying PGCCs and their progeny, characterizing the tumour microenvironment and uncovering mechanisms of therapy resistance. High-throughput platforms, such as Seq-Well and 10× Genomics, support large-scale single-cell RNA sequencing (scRNA-seq) studies. Analysing scRNA-seq data from cells exposed to potential anti-PGCC therapies can reveal affected pathways such as cell cycle regulation, metabolism and ferroptosis sensitivity, aiding in biomarker discovery and therapeutic development (Refs. 77, 78). However, there are significant hurdles to implementing PGCC identification with scRNA-seq, mostly due to multinucleation and polyploidy, which lead to misdiagnosis. The Scrublet and Doublet finder tools help solve these difficulties, though their sensitivity is limited (Ref. 79). Although scRNA-seq provides comprehensive transcriptional alterations, it does not capture tumour architectural localization information. Spatial transcriptomics permits PGCC localization in TME, revealing their location in relation to other cell types and tumour habitats. Furthermore, mathematical modelling can aid in understanding the behaviour and dynamics of the PGCC population in different tissue settings (Ref. 80).

To conclude, we provide a decision framework for the model selection as a practical guidance for the researcher to understand the PGCC biology. As shown in Figure 2, experimental models vary widely in throughput and biological relevance. High-throughput 2D cultures offer low fidelity but excellent reproducibility and cost-effectiveness, whereas 3D cultures provide moderate fidelity and diverse functional applications. In contrast, PDX models represent the highest fidelity system with low throughput and high cost.

## Clinical implications

PGCCs are a subset population implicated in therapy resistance, tumour progression, metastasis and recurrence associated with poor prognosis in various malignancies (Ref. 81). These cells retain near-complete viability even at high concentrations of paclitaxel and also at a weekly metronomic dose (Refs. 53, 82, 83), producing unique, resistant daughter cells that underscore the importance of understanding their biology, translational potential and therapeutic vulnerabilities. Furthermore, this condition was validated in various malignancies and in PDX models (Ref. 61), demonstrating varied sensitivity but consistent PGCC enrichment across models. Although PGCC development is influenced by various factors, including the diverse tumour niche in patient tumours, ex vivo studies indicate that paclitaxel therapy enhances PGCCs. Furthermore, clinical data verify and support that the presence of patient tissue with a high PGCC count is linked with a relatively unfavourable prognosis (Ref. 33). Including the prevalence data of PGCCs across different cancer types (Table 3) in your manuscript adds valuable clinical context and supports the significance of PGCCs as prognostic markers.

**Table 3.** Clinical prevalence of PGCCs across different cancer types

Cancer type	Sample used	Sample size	PGCCs prevalence (%)	HR* (95% CI)	*p*	Reference
Anal canal	Blood	*n* = 15	40	1.990 (1.087–3.644)	0.023	(Ref. 112)
Colon		*n* = 76	11.84			
NSCLC		*n* = 45	35.56			
Gastric		*n* = 51	17.65			
Breast		*n* = 29	13.79			
Kidney		*n* = 12	16.67			
Prostate cancer	Urine	*n* = 45	55.6	–	–	(Ref. 113)
Breast cancer	tumour	*n* = 52 (with and without metastasis)	High	–	0.000	(Ref. 114)
		*n* = 11 (benign)	0			
Primary ovarian tumour	tumour	*n* = 21(with metastasis)	85.71	–	0.000	(Ref. 115)
		*n* = 26 (without metastasis)	23.08			
Colorectal Cancer	tumour	*n* = 51 (well-differentiated CRC)	27.45	–	0.000	(Ref. 116)
		*n* = 56 (moderately differentiated CRC)	50			
		*n* = 51 (poorly differentiated CRC)	90.20			
Hepatocellular carcinoma	tumour	*n* = 56	35.7	–	–	(Ref. 117)
	tumour	*n* = 169	37	–	–	(Ref. 118)
	TCGA dataset	*n* = 350	64.8			
Angiosarcoma	tumour	*n* = 58	41.4	2.20 (1.17–4.15)	0.0142	(Ref. 119)

Abbreviations: TCGA: The Cancer Genome Atlas; CRC: colorectal cancer; NSCLC: non-small cell lung cancer. *HR: hazard ratio, CI: confidence interval.

Induced polyploidy via whole genome doubling (WGD) also correlates with poor prognosis and therapy resistance (Ref. 84). Clinically, their numbers increase in late-stage disease and post-chemotherapy. Specific molecular markers in PGCCs are associated with chemoresistance. They are also implicated in resistance to targeted therapies like PARP inhibitors (Ref. 84). Thus, PGCCs are a crucial predictor of treatment failure and disease recurrence, necessitating more focused therapeutics. Beyond resistance, PGCCs also drive tumour progression and metastasis by generating invasive progeny through asymmetric division and fostering cellular heterogeneity (Ref. 8). PGCCs promote local invasion and distant metastases, contributing to tumour recurrence. Their daughter cells can undergo EMT, enhancing motility and invasiveness (Ref. 85). In addition, PGCCs are also involved in vasculogenic mimicry and are more abundant in metastatic lesions (Ref. 86). They act as a reservoir of resistant cells, re-populating tumours after treatment and influencing the TME to favour progression and chemoresistance.

The presence of circulating tumour cells with increased genomic content (CTC-IGC) correlates with poorer progression-free survival in ⁓20% patient blood samples of prostate cancer with follow-up averaging ~45 months (Ref. 10). Their numbers correlate with tumour grade and stage and distinguish them prognostically from general CTCs, though they share stem cell-like traits. However, the exact biological equivalence and functional similarities between CTC-IGC and lab-generated PGCCs have not been thoroughly verified. The current view is that circulating PGCCs are a therapeutically significant subset that may overlap but differ from lab-induced PGCCs. Despite over 15 years of study, no PGCC-targeted therapeutics have entered clinical trials, largely due to the complex biology and the lack of robust model systems to fully examine them. Furthermore, creating tailored medicines is related to toxicity, seeking mitigation routes and focused delivery of pharmaceuticals, which hinder clinical application.

The current evidence suggests that PGCC detection is primarily a prognostic marker, associated with poor overall survival, regardless of stage, as indicated by substantial relationships in patient cohorts. However, PGCC enrichment after chemotherapy (e.g. paclitaxel) suggests a possible predictive role in therapeutic resistance (Ref. 87), yet this distinction is frequently muddled in the literature. in vitro colonization assays have demonstrated that PACC populations can regain proliferative ability in metastatic locations by displaying a PACC-specific partial epithelial-to-mesenchymal transition phenotype and a pro-metastatic secretory profile (Ref. 88). This suggests that the higher metastatic competence of PACC state cells was predictive of future metastases. As a result, defining PGCC as either prognostic or predictive is challenging, but clarifying this dual yet distinct value in the text is crucial.

## Standardization and reproducibility challenges in PGCC research

PGCC research currently suffers from significant reproducibility issues stemming from variable definitions and inconsistent methodologies. First, definitional variability is notable in size thresholds used to identify PGCCs, which range broadly from 15 to 50 μm across different studies (Ref. 89). This phenotypic heterogeneity complicates cross-study comparisons and meta-analyses. Following this, the timing of assessment post-treatment is often inconsistent, which could bias detection and measurement outcomes depending on cell cycle and treatment dynamics; culture conditions such as serum concentration, matrix stiffness and cell density vary widely, affecting PGCC induction, growth and functional characterization, while not exclusively on PGCCs, these timing inconsistencies and environmental factors influencing cell dynamics align with significant hurdles in PGCC induction and characterization in experimental settings (Ref. 90).

To address these challenges, consensus is needed on standardized PGCC definitions incorporating morphology and ploidy, harmonized protocols for timing post-treatment assessment and guidelines for controlled culture conditions. The adoption of batch effect controls and rigorous quantification criteria will improve reproducibility. Given the acknowledged terminological confusion and heterogeneity in PGCC research, we strongly recommend that the field adopt formal consensus guidelines detailing minimal criteria for PGCC identification, validation pipelines and reporting standards to bolster reproducibility and clinical applicability.

**Figure 3.**
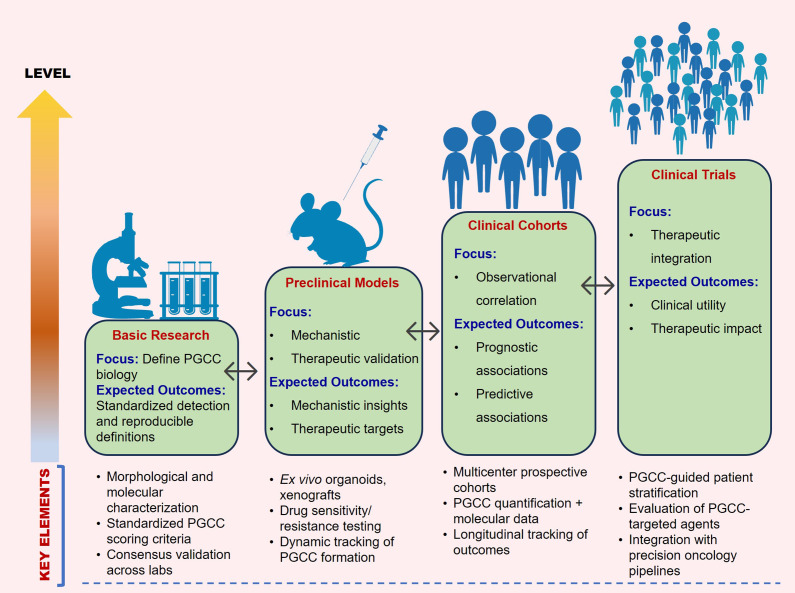
Translational framework for PGCC research. A stepwise pathway from defining PGCC biology and standardizing detection, through mechanistic and therapeutic validation in experimental models, to clinical correlation in multicentre cohorts, culminating in PGCC-guided patient stratification and targeted therapy development. Bidirectional arrows highlight the iterative nature of discovery and translation.

We propose a practical validation pipeline to support clinical translation of PGCC research. It combines standardized PGCC scoring using both morphology and ploidy measurements with prospective, multicentre testing. The framework also includes tracking patient outcomes over time and using unified data-reporting guidelines to reliably link PGCC features with treatment response and prognosis. Figure 3 provides an overview of the translational framework for PGCC research.

## Controversies and unresolved questions

The literature strongly supports the claim that PGCCs contribute to tumour growth and aggressiveness through the generation of progeny that defy current treatments. The majority of studies reveal PGCCs cellular plasticity by displaying a variety of treatment responses, which can lead to relapse and therapy resistance. In contrast, this malformed percentage of the population is responsible for terminal senescence and apoptosis. These non-proliferating cells exhibit passive metabolism, irreversible growth arrest and dormant rather than active progression. In some cellular circumstances, these cells were thought to be inactive and tumour suppressive. These divergence findings are based on research, such as models that rely on functional p53 status, which promotes polyploid cell senescence and death, whereas p53-deficient situations result in proliferation and the formation of resistant progeny. Furthermore, different experimental settings alter the fate of PGCC as it transitions from proliferative to senescent stages, suggesting that the TME influences physiological heterogeneity. Furthermore, approaches and detection methods for PGCC identification based on parameters such as morphological evaluation, molecular markers and genomic quantification influence the conclusion regarding the PGCCs origin and progeny. Finally, changes in the histology of diverse cancers cause mutations and genomic instability, which alter the dynamics of PGCC and complicate direct comparisons.

## Future directions and perspectives

Although major advances in experimental designs have been made to better understand the PGCC’s complicated biology, there are still gaps in understanding lineage plasticity, dynamic behaviour and TME interactions with current model systems. Upcoming research should employ integrative designs that combine modern in vitro systems with in vivo imaging to gain a deeper understanding of the processes. Organ-on-a-chip (OoC) devices are a game-changing technology for developing physiologically appropriate in vitro models, providing precise control over the cellular milieu and enabling the investigation of complicated cell–cell interactions inside the TME. OoCs allow for the integration of numerous cell types and provide the potential for personalized medicine by investigating individual tumour features and treatment responses utilizing patient-derived cell lines (Ref. 91). Furthermore, vascular interfaces and immune co-cultures can be replicated under physiological shear stress to investigate PGCC migration and communication with stromal cells (Ref. 92). In addition, these systems enable long-term live imaging, PGCC dormancy studies and the examination of secretory patterns, as compared to static organoid models. OoC systems can replicate tumour niches in 3D, but they lack controlled perfusion, dynamic flow and temporal modulation of microenvironmental stimuli. However, using these models will elucidate how physical and metabolic processes impact PGCC-driven outcomes.

The sensible use of new alternative methods (NAMs), such as patient-derived organoids (PDOs) and ex vivo models, is revolutionizing pre-clinical drug testing while decreasing dependence on animal models, which is also significant. The strategic use of NAMs to dissect molecular processes under specified settings can be handled adequately; nevertheless, some situations, such as involvement of PGCC in metastatic re-seeding or progeny lineage tracing, necessitate in vivo validation to capture the systemic and temporal complexity. Therefore, a tiered research strategy combining NAM-based predictive screening, multi-scale OoC modelling and selective in vivo confirmation is expected to speed up translation while reducing animal use.

Furthermore, the inability to trace the existence of PGCCs over time in live tissue represents a significant technical gap. The combination of intra-vital multiphoton microscopy and genetic barcoding techniques is a developing option that might enable the visualization of PGCC in a variety of contexts. Furthermore, using reporter structures to better understand PGCC metabolism and lineage identification within organoid systems might assist in linking in vitro and in vivo studies (Ref. 93). Furthermore, analysing complex data from PGCC models incorporating computational biology, bioinformatics and artificial intelligence (AI) will eventually allow the field to bridge descriptive observations with quantitative, mechanistic insights, bringing PGCC biology closer to therapeutic exploitation.

## Conclusion

PGCCs are now a crucial and intriguing part of cancer biology that requires reliable model systems for in-depth research. While advanced 3D models and co-culture systems offer increasingly complex platforms to replicate the tumour microenvironment and intercellular interactions regulating PGCC development and activity, *in* *vitro* 2D cultures offer simplicity and scalability for early characterization. The study of PGCCs in a living organism is facilitated by *in* *vivo* models, particularly *Drosophila* and xenograft models, which provide insight into the tumour’s growth, metastasis and resistance to treatment. Finally, by offering more clinically relevant systems for translational research, *ex* *vivo* models such as primary tumour-derived cultures, tumour explants and circulating tumour cells help close the gap between *in* *vitro* and *in* *vivo* investigations. Despite the advancements, the field still struggles to adequately encapsulate the diversity and dynamic character of PGCCs. Future studies should focus on developing more complex and integrated model systems that utilize microfluidic technology, patient-derived materials and cutting edge imaging methods. Such advancements will undoubtedly accelerate our understanding of PGCC biology and pave the way for novel therapeutic strategies targeting these elusive cells.
